# Comparative analysis of urinary continence recovery after open and laparoscopic radical prostatectomy: a retrospective cohort study

**DOI:** 10.25122/jml-2026-0004

**Published:** 2026-02

**Authors:** Alexandru-Ionuț Cherciu, Mihai-Cristian Persu, Andrei-Cosmin Bumbea, Mădălina-Maria Cherciu, Mihnea Cristian Firoiu, Radu Tiberiu Vrabie, Emilian Bolovan, Dragoș Mihail Arbunea, Darius Marian Brînzan, Andreea-Iuliana Ionescu, Ovidiu-Gabriel Bratu

**Affiliations:** 1 Department of Urology, Carol Davila University of Medicine and Pharmacy, Bucharest, Romania; 2 Department of Urology, The University Emergency Hospital Bucharest, Bucharest, Romania; 3 Department of Oncological Radiotherapy and Medical Imaging, Carol Davila University of Medicine and Pharmacy, Bucharest, Romania; 4 Department of Medical Oncology, Colțea Clinical Hospital, Bucharest, Romania

**Keywords:** urinary incontinence, radical prostatectomy, laparoscopic prostatectomy, continence recovery, functional outcomes

## Abstract

Urinary incontinence remains a major functional complication after radical prostatectomy. Although laparoscopic radical prostatectomy (LRP) provides perioperative advantages over open radical prostatectomy (ORP), its impact on continence recovery remains uncertain. This study aimed to compare urinary continence recovery at 3, 6, and 12 months after ORP and LRP and to explore clinical predictors of postoperative continence. This retrospective two-centre cohort included 75 consecutive patients undergoing ORP (*n* = 50) or LRP (*n* = 25) between January 2022 and December 2024, performed by the same surgical team. Continence was defined as 0–1 pad/day and assessed at predefined intervals. Between-group differences were expressed as absolute risk differences with 95% confidence intervals (CIs). Prespecified multivariable logistic regression models were constructed, with Firth penalized regression applied at 12 months due to sparse events. Baseline oncologic characteristics were comparable, with higher body mass index (BMI) in the ORP group (*P* < 0.001). LRP was associated with shorter operative time, lower blood loss, and reduced hospitalization (all *P* < 0.001). Continence rates did not differ significantly at 3 months (62% vs. 64%), 6 months (72% vs. 76%), or 12 months (86% vs. 88%). Surgical approach was not independently associated with continence at any time point, whereas higher BMI was consistently associated with persistent incontinence. Continence recovery was broadly similar between ORP and LRP. Despite perioperative advantages of LRP, functional outcomes were more strongly associated with patient-related factors than with surgical access. Larger prospective studies are warranted.

## Introduction

Urinary incontinence (UI) remains one of the most consequential functional complications following radical prostatectomy, with significant implications for postoperative quality of life, psychological well-being and satisfaction with oncologic treatment. The underlying mechanisms are multifactorial, typically involving damage to the sphincteric complex, disruption of pelvic floor support structures, periurethral fibrosis, and injury to autonomic and somatic innervation essential for continence control [1-3].

The introduction of minimally invasive prostatectomy—initially laparoscopic and later robot-assisted—has aimed to minimize soft-tissue trauma, enhance visualization of the prostatic apex, and refine dissection around continence-related structures. These technical advantages have led to the assumption that laparoscopic radical prostatectomy (LRP) may facilitate earlier continence recovery compared to traditional open radical prostatectomy (ORP) [4-7]. However, published data remain heterogeneous. While perioperative outcomes consistently favour minimally invasive surgery, long-term continence results tend to converge when nerve-sparing preservation, urethral length, and anastomotic quality are optimized [8-12].

Importantly, laparoscopic and robot-assisted radical prostatectomy are not interchangeable. Although both are minimally invasive, robotic platforms may introduce additional technical and ergonomic advantages that can influence functional outcomes; therefore, evidence generated predominantly in robot-assisted cohorts should not be assumed to apply directly to purely laparoscopic series [8-12].

Continence recovery is influenced by a complex interrelated set of factors, including age, BMI, prostate volume, preoperative urinary function, extent of nerve sparing, apical dissection technique, and general surgical expertise [13-17]. Understanding the relative contribution of surgical approach versus patient-specific characteristics is therefore crucial for preoperative counselling and overall management, clinical decision-making, and postoperative rehabilitation strategies [18].

In this context, we conducted a comparative analysis of continence recovery following ORP and LRP within a contemporary single-institution cohort. Our study integrates detailed perioperative evaluation, predefined continence endpoints, and multivariable analyses to examine whether surgical approach is associated with functional recovery.

## Material and Methods

### Study design and population

This retrospective cohort study included 75 consecutive patients who underwent radical prostatectomy for clinically localized prostate adenocarcinoma between January 2022 and December 2024 at two urology units: a public tertiary referral hospital and a private surgical clinic.

Although procedures were performed at two institutions, both centres functioned as parallel clinical settings in which the same surgical team operated throughout the study period. The operating surgeons were identical across both institutions and followed standardized surgical principles and perioperative management protocols. Consequently, differences between centres reflected administrative structures and patient referral pathways rather than variations in surgical personnel, technical execution, or operative philosophy.

Inclusion criteria comprised histologically confirmed localized prostate adenocarcinoma treated with open radical prostatectomy (ORP) or laparoscopic radical prostatectomy (LRP), with available follow-up data regarding continence status at 3, 6, and 12 months. Patients with prior pelvic radiotherapy, salvage procedures, preoperative urinary incontinence, or incomplete follow-up were excluded from analysis.

Selection of the surgical approach was based on shared decision-making between the patient and surgeon, institutional logistics, and operating room availability rather than on predefined oncologic risk stratification. Both surgeons routinely performed both ORP and LRP during the same period.

### Surgical technique and perioperative management

Radical prostatectomy was performed either through an open retropubic approach or a transperitoneal laparoscopic approach, according to surgeon and patient preference as previously described.

Pelvic lymph node dissection was performed based on oncologic risk stratification and contemporary guideline recommendations. The extent of lymphadenectomy (limited vs extended) was determined intraoperatively according to preoperative staging and risk classification.

Nerve-sparing was attempted whenever oncologically appropriate and was categorized intraoperatively as bilateral, unilateral, or non–nerve-sparing based on tumour characteristics and surgeon judgment. The decision to preserve neurovascular bundles was individualized and prioritized oncologic safety.

Bladder neck preservation was performed whenever feasible. Vesicourethral anastomosis was performed using a running suture technique in both ORP and LRP cases, following standardized institutional principles. Posterior musculofascial reconstruction (Rocco stitch) was performed selectively at the discretion of the operating surgeon.

A transurethral catheter was routinely maintained for approximately 7–10 days postoperatively, depending on intraoperative findings and cystographic evaluation when indicated.

All patients received standardized verbal counselling regarding pelvic floor muscle exercises following catheter removal. However, a formal structured pelvic floor rehabilitation program with supervised physiotherapy was not uniformly implemented during the study period.

Perioperative care protocols were otherwise comparable between the two institutions, including antibiotic prophylaxis, thromboprophylaxis, and postoperative follow-up scheduling.

### Continence assessment

Urinary continence was defined as the use of 0–1 pad per day, consistent with commonly adopted pragmatic definitions in postoperative functional assessment. Continence status was evaluated at routine outpatient follow-up visits scheduled at 3, 6, and 12 months after surgery.

Pad usage was documented based on patient self-report during clinical evaluation and recorded in standardized clinical notes. No validated questionnaires (such as EPIC-26 or ICIQ-UI SF) were systematically administered during the study period.

Because continence assessment relied on patient-reported pad usage and routine documentation rather than on structured questionnaire-based evaluation, a degree of measurement variability and potential misclassification cannot be excluded. However, follow-up visits were conducted by the same surgical team across both institutions, thereby minimizing inter-observer variability.

### Statistical analysis

Statistical analysis and graphical outputs were performed using JASP (Version 0.95.2), with penalized regression analyses conducted R. Continuous variables (age, BMI, PSA, prostate volume, PSA density, operative time, estimated blood loss, and length of hospitalization) were assessed for normality using the Shapiro–Wilk test. Because several variables showed significant deviation from normality, continuous data were summarized as mean ± standard deviation. Between-group comparisons (ORP vs. LRP) were performed using both Student’s *t*-test when assumptions were met, with Mann–Whitney U tests applied as non-parametric confirmation. Equality of variances was examined using Levene’s test.

Categorical variables (International Society of Urological Pathology [ISUP] grade group, clinical Tumor–Node–Metastasis [TNM] stage) were compared using Chi-square tests, with Fisher’s exact test applied when expected cell counts were insufficient. Frequencies and proportions were calculated for all categorical variables. Continence recovery was assessed at predefined follow-up intervals (3, 6, and 12 months). Because continence status was evaluated only at discrete timepoints, conventional time-to-event analysis was not considered appropriate due to interval censoring. Accordingly, recovery was defined as the proportion of patients who were continent at each follow-up visit. Between-group differences were expressed as absolute risk differences (LRP − ORP) with corresponding 95% confidence intervals. Group comparisons were performed using Fisher’s exact test.

To identify independent predictors of urinary continence at each follow-up interval (3, 6, and 12 months), multivariable logistic regression analyses were conducted. Two prespecified models were examined: an oncology model (including surgical approach, age, ISUP grade, and clinical stage), and a clinical model (including surgical approach, age, body mass index [BMI], and prostate volume). The dependent variable in each model was continence status (continent vs. incontinent).

Given the modest sample size and the limited number of incontinence events, particular attention was paid to events-per-variable considerations to reduce the risk of model overfitting. Therefore, the number of predictors included in each model was restricted a priori based on clinical relevance. Odds ratios (ORs) with 95% confidence intervals (CIs) were reported for all models.

At 12 months, near-complete continence rates resulted in sparse outcome events. Therefore, penalized logistic regression using Firth’s correction was applied to mitigate small-sample bias and potential separation. Model discrimination was evaluated using the area under the receiver operating characteristic curve (AUC), and explained variation was assessed using Nagelkerke’s R^2^. Estimates derived from the 12-month models were interpreted cautiously due to the limited number of events.

Prior to multivariable modelling, potential collinearity between perioperative variables (operative time, estimated blood loss, and length of hospitalization) was examined. Highly correlated variables were not entered simultaneously into the same regression model.

All statistical tests were two-sided, and a *P* value < 0.05 was considered statistically significant. Given sample size constraints, multivariable findings should be considered exploratory rather than confirmatory.

## Results

### Baseline characteristics

A total of 75 patients met inclusion criteria, including 50 who underwent ORP and 25 who underwent LRP. Baseline demographic and oncologic characteristics are summarized in Table 1.

**Table 1:** Baseline characteristics

Variable	ORP	LRP	*P* value
**Age (years)**	66.10 ± 741	64.56 ± 6.06	0.372
**BMI (kg/m2)**	27.39 ± 1.57	25.18 ± 2.02	< 0.001
**PSA (ng/mL)**	15.65 ± 12.71	13.79 ± 12.44	0.548
**PSA density**	0.273 ± 0.183	0.238 ± 0.147	0.416
**Prostate volume (mL)**	55.16 ± 14.09	54.92 ± 14.65	0.945
**ISUP grade**	Similar	similar	0.804
**cTNM stage**	Similar	similar	0.949

Patients in the ORP group had a higher BMI compared with the LRP group (mean ± SD: 27.39 ± 1.57 vs. 25.18 ± 2.02 kg/m^2^; *P* < 0.001). No statistically significant differences were observed between groups regarding age, PSA level, prostate volume, PSA density, ISUP grade group, or clinical TNM stage.

Oncological profiles were equivalent: the distribution of ISUP grade (*P* = 0.804) and clinical TNM stage (*P* = 0.949) showed no statistically significant differences between surgical approaches. These findings indicate well-balanced cancer characteristics and comparable baseline disease severity across groups.

### Perioperative outcomes

Significant perioperative differences were noted between techniques, as presented in Table 2. Compared with ORP, LRP was associated with reduced estimated blood loss (525 ± 116.6 vs. 392 ± 118.7 mL; *P* < 0.001) and shorter hospitalization (6.48 ± 1.05 vs. 4.92 ± 1.15 days; *P* < 0.001).

**Table 2:** Perioperative outcomes

Variable	ORP	LRP	*P* value
**Operative time (min)**	226.5 ± 28.0	189.0 ± 19.42	< 0.001
**Blood loss (mL)**	525 ± 116.6	392 ± 118.7	< 0.001
**Hospitalization (days)**	6.48 ± 1.05	4.92 ± 1.15	< 0.001

**Figure 1:**
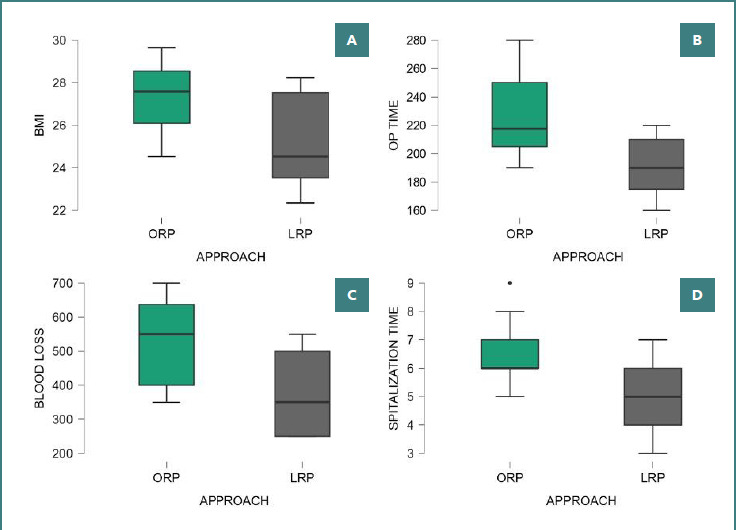
Perioperative outcomes comparing open radical prostatectomy (ORP) and laparoscopic radical prostatectomy (LRP). A, Body mass index (BMI); B, Operative time; C, Estimated blood loss; D, Hospitalization time.

There was also a significantly shorter operative time in the LRP group compared with ORP (226.5 ± 28.0 vs. 189.0 ± 19.42; *P* < 0.001). The distribution of perioperative variables is shown in Figure 1.

### Urinary continence outcomes (0–1 pad/day definition)

**Figure 2:**
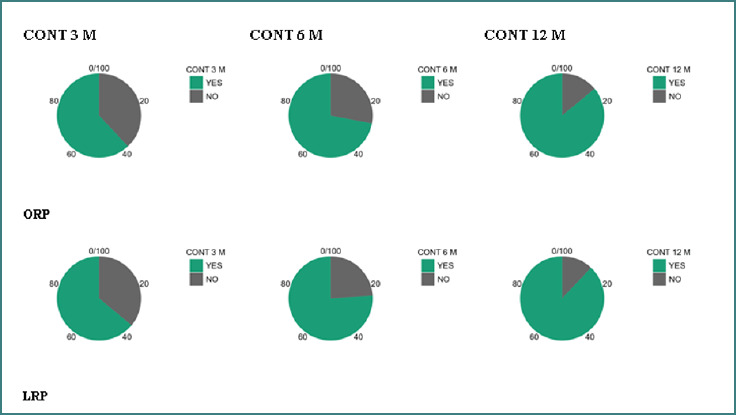
Continence rates at each time point (3, 6, 12 months) by surgical approach. ORP, open radical prostatectomy; LRP, laparoscopic radical prostatectomy.

At 3 months postoperatively, continence rates were comparable between the two groups (ORP: 62% vs. LRP: 64%; risk difference +2%; 95% CI, 21.1 to +25.1; Fisher’s exact *P* = 1.000). A similar pattern was observed at 6 months, with continence achieved in 72% of ORP patients and 76% of LRP patients (risk difference +4%; 95% CI,16.9 to +24.9; *P* = 0.788). By 12 months, continence rates improved substantially in both cohorts, reaching 86% for ORP and 88% for LRP (risk difference +2%; 95% CI, 14.0 to +18.0; *P* = 1.000). At no time point were statistically significant differences observed between surgical approaches, indicating that the choice of technique did not influence functional recovery. All findings are shown in Table 3 and illustrated graphically in Figure 2.

**Table 3:** Continence recovery by timepoint

Timepoint	ORP *n/N* (%)	LRP *n/N* (%)	Risk difference (LRP−ORP), %	95% CI	*P* value (Fisher)
3 months	31/50 (62%)	16/25 (64%)	+2	−21.1 to +25.1	1.000
6 months	36/50 (72%)	19/25 (76%)	+4	−16.9 to +24.9	0.788
12 months	43/50 (86%)	22/25 (88%)	+2	−14.0 to +18.0	1.000

### Multivariable analysis

Multivariable logistic regression analyses are presented in Tables 4 and 5. In the oncology models (Table 4), surgical approach was not independently associated with incontinence at 3, 6, or 12 months. At 12 months, increasing age was independently associated with higher odds of persistent incontinence in the penalized model, whereas ISUP grade and clinical stage were not significant predictors.

**Table 4:** Multivariable oncology models for incontinence

Variable	3 Months OR (95% CI)	*P*	6 Months OR (95% CI)	*P*	12 Months OR (95% CI)*	*P*
Surgical approach (LRP vs ORP)	0.94 (0.34–2.60)	0.902	0.88 (0.27–2.84)	0.827	1.10 (0.24–4.44)	0.892
Age (per year)	1.02 (0.95–1.10)	0.543	1.08 (0.99–1.17)	0.094	1.11 (1.00–1.25)	0.044
ISUP (Low vs High)	1.27 (0.48–3.32)	0.633	2.59 (0.85–7.95)	0.095	1.14 (0.29–4.47)	0.848
Clinical stage (T1 vs ≥T2)	1.80 (0.59–5.49)	0.298	1.55 (0.46–5.21)	0.482	1.08 (0.21–4.45)	0.919

* 12-month model estimated using Firth penalized logistic regression.

Model performance:

3M AUC ≈ 0.61 (Nagelkerke R^2^ ≈ 0.04)

6M AUC ≈ 0.71 (Nagelkerke R^2^ ≈ 0.13)

12M (Firth) moderate discrimination

**Table 5:** Multivariable clinical models for incontinence

Variable	3 Months OR (95% CI)	*P*	6 Months OR (95% CI)	*P*	12 Months OR (95% CI)*	*P*
Surgical approach (LRP vs ORP)	2.10 (0.61–7.25)	0.239	2.02 (0.52–7.87)	0.309	3.67 (0.61–24.07)	0.151
Age (per year)	1.01 (0.94–1.09)	0.821	1.04 (0.96–1.14)	0.322	1.05 (0.94–1.19)	0.356
BMI (per kg/m^2^)	1.46 (1.07–1.99)	0.017	1.54 (1.07–2.21)	0.020	2.34 (1.34–5.31)	0.001
Prostate volume (per mL)	1.00 (0.96–1.03)	0.807	1.02 (0.98–1.06)	0.418	1.05 (1.00–1.11)	0.073

* 12-month model estimated using Firth penalized logistic regression.

Model performance:

3M AUC ≈ 0.69 (Nagelkerke R^2^ ≈ 0.13)

6M AUC ≈ 0.73 (Nagelkerke R^2^ ≈ 0.18)

12M AUC ≈ 0.90 (interpret cautiously due to sparse events)

In contrast, the clinical models (Table 5) demonstrated a consistent association between body mass index and continence recovery. Higher BMI was independently associated with increased odds of incontinence at 3 months (*P* = 0.017), 6 months (*P* = 0.020), and 12 months in the Firth penalized model (*P* = 0.001). The magnitude of this association increased over time. Surgical approach remained non-significant across all models and time points. Prostate volume showed a borderline association at 12 months but did not reach statistical significance.

The estimates for surgical approach were characterized by wide confidence intervals, particularly in the 12-month models, reflecting limited precision due to the small number of late incontinence events. Overall, these findings indicate that patient-related factors — most notably BMI — rather than surgical technique, were the primary determinants of continence recovery.

## Discussion

In this retrospective two-centre cohort, we compared urinary continence recovery following open radical prostatectomy and laparoscopic radical prostatectomy. Although procedures were performed in two distinct institutional settings, surgeries were conducted by the same surgical team during the same period, thereby minimizing variability related to surgeon experience or operative philosophy. Perioperative outcomes favoured LRP in terms of operative time, estimated blood loss, and hospitalization duration, whereas continence recovery trajectories at 3, 6, and 12 months were broadly comparable between approaches.

At early follow-up intervals, multivariable models suggested an association between laparoscopic approach and higher odds of continence recovery. However, effect estimates were large and accompanied by wide confidence intervals, reflecting the limited number of incontinence events and potential model instability. Given events-per-variable constraints and the modest sample size, these findings should be interpreted as exploratory rather than confirmatory.

Importantly, early differences in continence recovery may be partially explained by perioperative factors. In our cohort, LRP was associated with significantly shorter operative time and lower blood loss. Reduced surgical trauma and improved visualization inherent to minimally invasive techniques may contribute to earlier functional recovery. However, when long-term continence at 12 months was considered, between-group differences diminished, suggesting that early advantages may not necessarily translate into sustained superiority.

The role of surgical approach in continence recovery remains debated. While minimally invasive techniques are often presumed to facilitate faster recovery, continence outcomes are strongly influenced by patient-related factors and detailed surgical execution, including nerve-sparing status, preservation of urethral length, bladder neck management, and anastomotic quality. In the absence of systematic data on these technical variables, surgical approach may act as a surrogate marker rather than an independent determinant of functional recovery.

Body mass index (BMI) demonstrated associations with continence in early models. However, baseline BMI differed between groups, and its effect direction should be interpreted cautiously considering potential residual confounding. Obesity is known to increase surgical complexity, and imbalance in baseline characteristics may partially explain observed associations.

This study has several limitations. First, the retrospective design introduces inherent risks of selection bias and confounding by indication. Although the study was conducted across two institutions, both functioned as parallel clinical settings in which the same surgical team performed procedures using standardized operative principles. Nevertheless, surgical approach was not randomized, and residual confounding cannot be excluded. Second, detailed intraoperative variables known to influence continence—such as nerve-sparing extent, posterior reconstruction consistency, and baseline urinary function—were not systematically captured. Third, continence was defined pragmatically as 0–1 pad per day and assessed through routine clinical documentation rather than validated questionnaires, which may introduce measurement variability. Additionally, continence was evaluated at discrete timepoints, limiting precision in time-to-recovery assessment.

Despite these limitations, the study provides a real-world comparison of ORP and LRP within a contemporary institutional setting, with standardized perioperative care and consistent follow-up.

Overall, our findings suggest that while LRP was associated with favourable perioperative parameters, continence recovery at 3, 6, and 12 months appeared broadly similar between approaches. Larger prospective studies incorporating standardized functional assessment and detailed surgical variables are required to clarify the independent contribution of surgical technique to continence outcomes.

## Conclusion

In this retrospective two-centre cohort study, urinary continence recovery at 3, 6, and 12 months following radical prostatectomy appeared broadly similar between open and laparoscopic approaches. Although laparoscopic surgery was associated with more favourable perioperative parameters, early differences in continence outcomes should be interpreted cautiously considering potential confounding and limited sample size.

These findings suggest that functional recovery is likely influenced by a complex interplay of patient-related and technical factors rather than by the surgical approach alone. Larger prospective studies incorporating standardized functional assessment and detailed surgical variables are needed to further clarify determinants of postoperative continence recovery.

## Data Availability

Further data is available from the corresponding author upon reasonable request.
